# Increased Rate of CD4+ T-Cell Decline and Faster Time to Antiretroviral Therapy in HIV-1 Subtype CRF01_AE Infected Seroconverters in Singapore

**DOI:** 10.1371/journal.pone.0015738

**Published:** 2011-01-27

**Authors:** Oon Tek Ng, Li Lin, Oliver Laeyendecker, Thomas C. Quinn, Yong Jiang Sun, Cheng Chuan Lee, Yee Sin Leo

**Affiliations:** 1 Division of Infectious Diseases, Johns Hopkins School of Medicine, Baltimore Maryland, United States of America; 2 Department of Infectious Disease, Tan Tock Seng Hospital, Singapore, Singapore; 3 Laboratory of Immunoregulation, Division of Intramural Research, National Institute of Allergy and Infectious Diseases, National Institutes of Health, Baltimore, Maryland, United States of America; McGill University, Canada

## Abstract

**Background:**

It remains controversial as to whether HIV-1 subtypes influence disease progression. Singapore offers a unique opportunity to address this issue due to the presence of co-circulating subtypes. We compared subtype CRF01_AE and non-CRF01_AE infected patients, with regards to estimated annual rate of CD4+ T-cell loss and time from estimated data of seroconversion (EDS) to antiretroviral therapy (ART).

**Methods:**

We recruited ART-naive patients with known dates of seroconversion between October 2002 and December 2007 at the Singapore Communicable Disease Centre, the national reference treatment centre. Multilevel mixed-effects models were used to analyse the rate of CD4+ T-cell decline. Time from EDS to ART was analyzed with the Kaplan-Meier survival method and compared with Cox proportional hazards models.

**Results:**

54 patients with previously assigned HIV-1 subtypes (24 CRF01_AE, 17 B, 8 B', 1 CRF33_01B, 3 CRF34_01B and 1 G) were observed for 89 patient-years. Subtype CRF01_AE and non-CRF01_AE infected patients did not differ in age, gender, risk factor, rate of symptomatic seroconversion, baseline CD4+ T-cell count, log_10_ viral load or haemoglobin concentration. The estimated annual rate of CD4+ T-cell loss was 58 cells/mm^3^/year (95% CI: 7 to 109; *P* = 0.027) greater in subtype CRF01_AE infected patients compared to non-CRF01_AE patients, after adjusting for age, baseline CD4+ T-cell count and baseline log_10_ viral load. The median time from EDS to ART was 1.8 years faster comparing CRF01_AE to non-CRF01_AE infected patient with a 2.5 times (95% CI: 1.2-5.0; *P* = 0.013) higher hazard for ART initiation, after controlling for age, baseline CD4+ T-cell count and baseline log_10_ viral load.

**Conclusions:**

Infecting subtype significantly impacted the rate of CD4+ T-cell loss and time to treatment in this cohort. Studies to understand the biological basis for this difference could further our understanding of HIV pathogenesis.

## Introduction

The global HIV-1 pandemic is characterized by extensive genetic diversity [1]. Uncertainty exists as to whether different subtypes manifest different pathogenicity, and by implication different host-pathogen interactions [2]. Genotypic differences in viral pathogenicity have implications in determining viral genotypic motifs important to disease progression, vaccine development and disease transmission [3]. Clinical guidelines may have to be subtype-specific if significant differences exist [4], [5]. Currently, the overwhelming majority of data on HIV-1 is based on subtype B, the predominant subtype in developed countries. However, subtype B accounts for only 10% of infections worldwide [2].

Two recent studies from northern Thailand with cohorts of subtype CRF01_AE infected patients reported a 3-year shorter median survival compared to age-matched individuals for the Western CASCADE cohort, where subtype B predominates [6], [7], [8]. This reduced survival could have been due to viral, host or environmental differences between the Thai and Western cohorts.

In Singapore, subtypes CRF01_AE and B exist in near equal proportions [9]. Patients treated at our centre have regular CD4+ T-cell count monitoring. This unique setting enabled us to study subtype differences in disease progression and corresponding clinical implications. The primary objective of our study was to determine the difference between subtype CRF01_AE and other circulating subtypes in the pre-ART annual rate of CD4 decline in subjects with known dates of seroconversion. Furthermore, we examined the time from infection to ART initiation by infecting subtype.

## Methods

### Study population

Details of the study participants and laboratory protocols have been previously published [9]. In brief, patients identified as seroconverters within a prior 2-year window by treating physicians at the Communicable Disease Centre from October 2002 to December 2007 were recruited. Inclusion criteria were either: (1) a previous negative HIV antibody test within 24 months of study entry followed by a positive test; (2) an indeterminate Western blot within 24 months of study entry followed by a positive test; (3) clinical syndrome consistent with seroconversion illness following recent risk factor for HIV-1 exposure with prior negative HIV antibody test within 24 months of study entry. After obtaining written consent, blood drawn for HIV-1 genotyping was centrifuged to obtain plasma samples stored at −80°C. Follow-up was per routine clinical care, where patients were seen at 3 to 6 monthly intervals with standard of care interventions provided following recognized guidelines [10], [11].

Clinical data was obtained from chart review. Only patients with 2 or more CD4+ T-cell counts measurements from at least 3 months following EDS were included in the current study. The 3-month interval was chosen to exclude results from the period of wide variability in CD4+ T-cell counts during recent seroconversion. Patients were followed until mid-May 2010 via chart review or censored either at ART initiation or loss to follow up (defined as more than 1 year without a clinic visit).

Informed written consent was obtained from all participants. The National Healthcare Group ethics committee approved this study and its guidelines were followed in the conduct of this study.

### Laboratory analysis

As published, HIV-1 pol gene sequences were previously determined using the Celera Diagnostics ViroSeq HIV-1 Genotyping System (version 2.0) (Celera Diagnostics, USA). Phylogenetic analysis for sub-typing against reference sequences from the Los Alamos HIV Sequence Database has been published [9]. CD4+ T-cell counts were analysed on the Becton Dickinson FACSCalibur analyser (Becton Dickinson International, Belgium). Plasma viral load was determined by the AMPLICOR HIV-1 MONITOR version 1.5 (Roche Molecular Systems, USA) before May 2007. After May 2007, the Abbott RealTime HIV-1 assay (Abbott Molecular Inc, USA) was used.

### Statistical analysis

The estimated date of seroconversion (EDS) was defined in order of priority as: (1) date of onset of clinical seroconversion syndrome, (2) date of indeterminate Western blot or (3) midpoint between date of last negative and first positive HIV-1 serology. Baseline CD4+ T-cell count was defined as the first result available at least 3 months from EDS. Viral loads in copies/ml were log_10_-transformed and those above the detectable limit were given the value equivalent to 750,000 copies/ml, the upper limit of the Roche assay. No result exceeded the detectable limit of the Abbott assay of 10 million copies/ml. The mode of transmission was divided into 5 categories (heterosexual, homosexual, bisexual, injection drug use and unknown). HIV-1 subtypes were dichotomised into subtype CRF01_AE and non-CRF01_AE. Baseline characteristics were compared between the subtype groups by Student's t-test and Wilcoxon Rank-Sum test for continuous variables and Fisher's exact test for categorical variables.

Multilevel linear mixed-effects models with unstructured covariance were used to estimate the annual rate of CD4+ T-cell decline [12]. The final model adjusted for age, baseline CD4+ T-cell count and baseline log10-transformed viral load. Presence of symptomatic seroconversion, mode of transmission and baseline haemoglobin level were dropped from the final model, as they did not improve model fit. As previous investigators used square-root transformation of CD4+ T-cell count to stabilize variance, the final model was repeated with transformation to ensure inferences were unaltered [4], [13].

The Kaplan-Meier survival method was used to examine progression from EDS to ART initiation. Cox proportional hazards models were used to estimate crude and adjusted hazard ratios for this endpoint. All analyses were performed using Stata 11 (Stata Corporation, USA), and all tests were conducted as 5% significance level.

## Results

### Population characteristics

54 patients met the inclusion criteria. Subtype distribution was as follows: 24 CRF01_AE, 17 B, 8 B', 1 CRF33_01B, 3 CRF34_01B and 1 G. The median age was 30.6 years (range: 17.2 to 57.9 years). The majority of patients were men (98%) and of Chinese ethnicity (96%). Risk factor for HIV-1 acquisition was homosexual exposure in 27 patients (50%), heterosexual in 13 patients (24%), bisexual in 7 patients (13%), intravenous drug use (IDU) in 1 (2%) patient and 6 (11%) patients with no self-reported risk. 50% of the patients had a history of symptoms consistent with seroconversion illness.

The mean baseline CD4+ T-cell count was 408 cells/mm3 (s. d.: 197 cells/mm3) with a median time from EDS to baseline count of 6.4 months (IQR: 4.8 to 7.9 months). Mean baseline log10-transformed viral load was 4.8 (s. d.: 0.76) obtained at median time from EDS of 5.8 months (IQR: 3.3 to 10 months).

The total observation time was 89 patient years. The median follow-up time from baseline CD4+ T-cell count to last recorded pre-ART CD4+T-cell count was 1.8 years (IQR: 1 to 2.3 years). 6 patients (2 CRF01_AE and 4 non-CRF01_AE) defaulted follow up after a median duration of 1.1 years (IQR: 0.7 to 2.1 years). There were no statistically significant demographic or baseline laboratory test differences between the two subtype groups (Table 1).

**Table 1:** Characteristics of patients by HIV-1 subtype^a^.

	Subtype CRF01_AE (n = 24)	Non-CRF01_AE (n = 30)	P-value
Age in years (median,IQR)	34.7 (27.4–42.3)	30.4 (27.5–37.0)	0.338
Male (number, column %)	23 (95.8)	30 (100)	0.444
Mode of transmission (number; column %)			
Heterosexual	7 (29)	6 (20)	0.813
Homosexual	11 (46)	16 (53)	
Bisexual	3 (13)	4 (13)	
IDU	1 (4)	0 (0)	
Unknown	3 (13)	4 (13)	
Symptomatic seroconversion (number, column %)	13 (54)	14 (47)	0.392
Baseline CD4+ T-cell count (cells/mm3) (mean, sd)	406 (209)	411 (191)	0.928
Baseline log_10_ transformed viral load (median, IQR)	4.97 (4.39–5.70)	4.71 (4.31–5.23)	0.172
Baseline haemoglobin (g/dL) (mean, sd)	14.0 (1.5)	14.4 (1.2)	0.318
Estimated date of seroconversion (EDS) (median, IQR)	Jan 2007 (Mar 2006 to April 2007)	December 2006 (October 2005 to Mar 2007)	0.741
Months from EDS to baseline CD4 (median, IQR)	6.3 (3.5–6.8)	6.5 (5.1–8.7)	0.304
Months from EDS to baseline viral load (median, IQR)	4.5 (3.1–10.3)	6.1 (3.5–12.2)	0.309
Median number of CD4 counts per patient (median, IQR)	6 (4–7)	6 (4–7)	0.604
Number initiated treatment (number, column %)	19 (79)	14 (47)	0.024
CD4+ T-cell count at treatment initiation (number, column %)			
<250 cells/mm^3^	13 (68)	7 (50)	0.273
250–350 cells/mm^3^	5 (26)	7 (50)	
>350 cells/mm^3^	1 (5)	0 (0)	

a Statistical tests as detailed in methods section.

### Rate of CD4+ T-cell Decline

The overall average rate of CD4+ T-cell decline without regard to subtype was 56 cells/mm^3^ per year (95%CI: 30 to 82 cells/mm^3^), controlling for age, baseline CD4+ T-cell count and log_10_ viral load. In the multilevel model including subtype, CRF01_AE infected patients had an average decline of 90 cells/mm3 per year (95%CI: 50 to 131 cells/mm3) compared to 32 cells/mm^3^ per year for non-CRF01_AE infected patients (95%CI: 1 to 64 cells/mm3), after controlling for age, baseline CD4+ T-cell count and log_10_ viral load. The difference in average yearly decline between the two subtype categories was statistically significant (P = 0.027) (Table 2). Square-root transformation of CD4+ T-cell count did not change the study inference; the rate of decline for subtype CRF01_AE infected patients compared to other subtypes remained significantly higher after adjusting for age, baseline CD4+ T-cell count and log_10_ viral load (-1.83/year; 95%CI: 0.27 to 3.40; P = 0.022).

**Table 2:** Independent predictors of rate of CD4 decline^a^.

	Difference in annual rate of CD4+ T-cell decline (cells/mm3/year)	95% Confidence Interval	P-value
Subtype (CRF01_AE vs non-CRF01_AE)	–58	–7 to –109	0.027
Baseline CD4+ T-cell count (per 100 cells/mm3 increase)	–21	–8 to –34	0.001
Baseline log_10_ viral load (per 1 log increase)	–32	–64 to 1	0.060

a Co-variates in the model were subtype, baseline CD4+ T-cell count, baseline log_10_ viral load and age. Baseline log_10_ viral load was borderline statistically significant.

### Time from EDS to ART initiation

**Figure 1:**
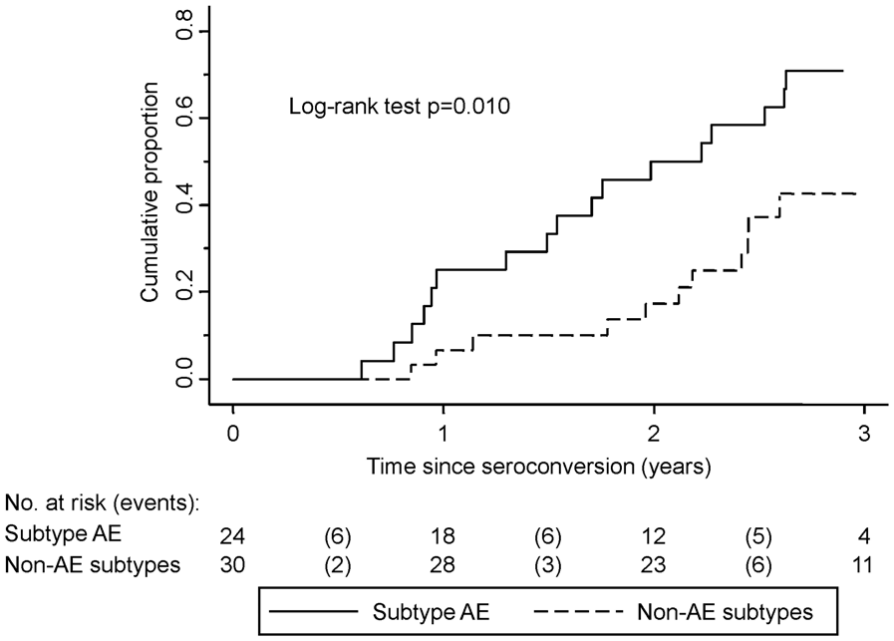
Cumulative proportion of HIV-1 patients initiating antiretroviral therapy by infecting subtype.

A greater proportion of CRF01_AE infected patients initiated ART (79% compared to 47%; P = 0.024) with similar CD4+ T-cell counts at ART initiation in the two groups (Table 1). The median time to treatment was 2.0 years (95% CI: 1.3-2.6) for CRF01_AE infected patients compared to 3.8 years (95% CI lower limit: 2.4) for non-CRF01_AE infected patients (Figure 1). Using Cox proportional hazards, the unadjusted hazard ratios comparing CRF01_AE to non-CRF01_AE infected patients was 2.5 (95%CI: 1.2 to 5.0; P = 0.013). After adjusting for age, baseline CD4+ T-cell count and log_10_ viral load, the HR was unchanged at 2.5 (95%CI: 1.2 to 5.1; P = 0.015).

## Discussion

In this cohort of seroconverters from Singapore, subtype status was strongly correlated with rate of CD4+ T-cell loss. Subtype CRF01_AE infected patients had a mean 58 cell/mm^3^/year greater adjusted loss of CD4+ T-cells compared to other infecting subtypes (mainly B) in our study. The resultant clinical outcomes were markedly different in that the median time to treatment initiation was 1.8 years faster in subtype CRF01_AE infected patients.

Differences in subtype progression have been documented in Africa [14], [15]. A recent study examining the differences in CD4+ T-cell loss between subtypes in Uganda reported a 73.7 cell/mm^3^ greater yearly loss in subtype D compared to subtype A infected patients [16]. In Southeast Asia, 2 studies from subtype CRF01_AE infected patients in Thailand reported shorter median survival times of 3 years compared to age-matched patients from Western cohorts, where subtype B predominates [6], [7]. This comparison was potentially confounded by inter-geographic differences in host genetics or access to care [4]. Our study was conducted in an overwhelmingly male Chinese population with universal access to healthcare [17]. There was no detectable baseline host difference between the co-circulating subtypes that would explain the phenotypic differences. Our results provide supportive evidence that infection with a CRF01_AE subtype contributed to the decreased survival reported from Thailand.

A study of injecting heroin users in Bangkok reported higher viral loads in subtype CRF01_AE compared to subtype B infected patients up to 9 months from seroconversion. However, no difference was documented in the rate of decline of CD4+ T-cell counts up to 2 years following seroconversion between CRF01_AE and B infected individuals [18]. Our study population compared to the Bangkok cohort had different risk profiles and potentially different access to medical care. In addition, both our study and the Bangkok cohort did not have data on host genetics, an interesting area of future research.

A recent study from Canada demonstrated longer time to treatment initiation among non-subtype B (mainly C, CRF02_AG and A) infected patients compared to subtype B [4]. In our study, CRF01_AE compared to B infected patients had a shorter time to ART initiation. If confirmed in larger cohorts, subtype status could contribute to clinical prognostication.

In conclusion, we document a marked difference in CD4+ T-cell decline and time to treatment comparing CRF01_AE and non-CRF01_AE HIV-1 infection in Singapore. Larger studies to confirm this result are planned. If confirmed, studies to understand the biologic basis for these differences could bear fruit to further our understanding of HIV-1 pathogenesis.
